# SAMPL7 protein-ligand challenge: A community-wide evaluation of computational methods against fragment screening and pose-prediction

**DOI:** 10.1007/s10822-022-00452-7

**Published:** 2022-04-15

**Authors:** Harold Grosjean, Mehtap Işık, Anthony Aimon, David Mobley, John Chodera, Frank von Delft, Philip C Biggin

**Affiliations:** 1 Structural Bioinformatics and Computational Biochemistry, Department of Biochemistry, South Parks Road, OX1 3QU Oxford, UK; 2 Diamond Light Source Ltd, Harwell Science and Innovation Campus, OX11 0QX Didcot, UK; 3 Computational and Systems Biology Program, Sloan Kettering Institute, Memorial Sloan Kettering Cancer Center, 10065 New York, NY USA; 4 Department of Pharmaceutical Sciences, Department of Chemistry, University of California, 92617 Irvine, California USA; 5 Research Complex at Harwell, Harwell Science and Innovation Campus, OX11 0FA Didcot, UK; 6 Centre for Medicines Discovery, University of Oxford, Old Road Campus, Roosevelt Drive, OX3 7DQ Headington, UK; 7 Structural Genomics Consortium, University of Oxford, Old Road Campus, Roosevelt Drive, OX3 7DQ Headington, UK

**Keywords:** Pleckstrin-homology domain interacting protein, Bromodomain, Fragment-based drug design, Crystallography, High-throughput screening, SAMPL challenge

## Abstract

A novel crystallographic fragment screening data set was generated and used in the SAMPL7 challenge for protein-ligands. The SAMPL challenges prospectively assess the predictive power of methods involved in computer-aided drug design. Application of various methods to fragment molecules are now widely used in the search for new drugs. However, there is little in the way of systematic validation specifically for fragment-based approaches. We have performed a large crystallographic high-throughput fragment screen against the therapeutically relevant second bromodomain of the Pleckstrin-homology domain interacting protein (PHIP2) that revealed 52 different fragments bound across 4 distinct sites, 47 of which were bound to the pharmacologically relevant acetylated lysine (Kac) binding site. These data were used to assess computational screening, binding pose prediction and follow-up enumeration. All submissions performed randomly for screening. Pose prediction success rates (defined as less than 2 Å root mean squared deviation against heavy atom crystal positions) ranged between 0 and 25% and only a very few follow-up compounds were deemed viable candidates from a medicinal-chemistry perspective based on a common molecular descriptors analysis. The tight deadlines imposed during the challenge led to a small number of submissions suggesting that the accuracy of rapidly responsive workflows remains limited. In addition, the application of these methods to reproduce crystallographic fragment data still appears to be very challenging. The results show that there is room for improvement in the development of computational tools particularly when applied to fragment-based drug design.

## Introduction

Fragment-based drug design (FBDD) has emerged as a successful field within the last two decades yielding several Food and Drug Administration (FDA) approved drugs, many more clinical candidates and a significant number of chemical probes. FBDD relies on the identification of low molecular weight molecules, typically between 150 and 350 Da, that bind to a protein target at a low affinity, ranging from low millimolar to low micromolar. Hits are normally identified through a biophysical assay that provides starting material for the chemical elaboration of the fragments into more potent compounds [1]. Throughput is an important aspect in FBDD as screening a larger quantity of fragments increases the number of hits identified therefore providing a larger number of opportunities for follow-up and optimization. The requirement for high-throughput means some of the slower biophysical approaches such as surface plasmon resonance (SPR), isothermal titration calorimetry (ITC) or nuclear magnetic resonance (NMR) spectroscopy are optimal methods in this scenario. In addition, these methods tend to be limited by the solubility of the fragment because large quantities of the (low affinity) fragment must be dissolved and exposed to the target to obtain a reliable biophysical signal.

X-ray crystallography has emerged as a leading technique in FBDD as it allows higher throughput and sensitivity than other biophysical fragment hit identification methods [2]. Multiple crystallographic fragment screening facilities have been created around the world and are based within or near synchrotrons to facilitate access to beamlines. The XChem, which is located within The Diamond Light Source (Didcot, U.K), is one of the world’s leading facilities and provides an automated and high-throughput crystallographic fragment screening pipeline with unprecedented speed and sensitivity. A highly reproducible and well-diffracting crystal system must first be obtained [3], then fragments dissolved in organic solvent are acoustically dispensed into the wells where suitable crystals have been observed [4]. Crystal harvesting is helped by the use of a shifter [5] and the resulting samples are shot in one of the beamlines equipped with robotic arms that facilitate automatic X-ray diffraction experiments leading to the acquisition of hundreds of electron density maps.

The low affinity of the fragments for the target implies that most proteins within the crystal are not fragment-bound, which results in no or incomplete electron density maps and therefore prohibits proper model building. The Pan-Dataset Density Analysis (PanDDA) takes advantage of the high number of electron density maps by creating a ground-state model that can be seen as an average electron density map from which outliers can be readily identified [6]. Since the majority of the datasets do not harbour fragment binding events, the ground state model will be representative of the unbound state and the identified outliers of the fragment-bound state. Subtraction of the ground-state from the electron density maps of outliers produces event maps that reveal additional densities that can be used to further guide crystallographic model building and/or complement traditional electron density maps. This process yields high-resolution fragment-bound 3D structures that can later be employed in the process of fragment elaboration.

Computational modelling is now an essential and omnipresent component of the whole drug discovery pipeline. This is also true for fragment-hit identification and hit-to-lead development where expensive screening experiments are now being replaced by modelling procedures that have incomparably higher throughput. The generated hypothesizes are then validated experimentally therefore offering a cheaper alternative to a purely laboratory-based workflow. Computational approaches also have the advantage of not being constrained by experimental limitations such as ligand solubility and synthesizability. Typically, computational drug discovery campaigns start with the screening of a large library of compounds against a target receptor with the aim of identifying a pool of best molecule candidates. This step normally relies on computationally cheap methods that can go through large libraries in a minimum amount of time [7]. Experimental data can also be incorporated in the workflow to guide the predictions. For example, crystallographic fragment screening identifies binders, and the associated bound structures can be used to constrain docking or generate pharmacophores.

In the early 2000s, much effort was dedicated to screening drug-like molecules as might be done in a drug re-positioning campaign. However, this suffers from the fact that the compounds composing the library may not be specific enough for the target and this may be particularly true for previously unexplored types of binding sites [8]. Furthermore, the smaller size of fragments means they are able to explore a much larger range of chemical space compared to larger molecules, which are more constrained by the binding site geometry. However, computational screening with fragments presents different challenges. Fragments have reduced chemical complexity and potency and therefore higher similarity between them when compared with larger drug-like molecules [9]. This implies that discriminating binders from non-binders or stronger from weaker binder is likely to be even more error prone. In addition, there is the complication that fragments can sometimes change their binding poses as chemical elaboration is performed. Whether this occurs or not depends on the result trade-off between enthalpy and entropy contributions as the fragment is expanded. This of course adds another layer of complexity to structure-based elaborations [10].

The Statistical Assessment of Proteins and Ligands (SAMPL) challenges are a series of computational prediction trials where participants are given the task of predicting the results of experiments in a fully blinded fashion. Thus, the true predictive power of the submitted methods is assessed in a prospective, unbiased manner. The participants come from both industrial and academical settings, and the nature of the tasks varies but they are always related to computer-aided drug design (https://www.samplchallenges.org/). The SAMPL challenges have been running since 2008 and many physico-chemical properties have been examined over the years, including the solvation free energies of small molecules [11], partition coefficients, [12–14] distribution coefficients [12, 15] and pKa predictions [12, 16, 17] or host-guest system binding affinities [18, 19]. Early SAMPL challenges (1 and 2) were not reported on, but later SAMPL challenges have been summarized for the community to help direct future research in these areas. SAMPL3 involved fragment screening and binding affinity predictions against trypsin where good enrichments and correlations were obtained for these tasks respectively [11]. SAMPL4 was divided into 3 stages: small molecule virtual screening, binding pose and binding affinity predictions which were carried out against the catalytic core domain of the HIV integrase [20]. This system has 3 different binding sites: the Y3 site, the Fragment and the LEDGF pockets. This added another difficulty to the predictions since participants had to determine which molecule binds to which site [21]. Overall, all 3 stages appeared quite challenging, although some methods achieved good enrichment for screening and good binding pose prediction. Binding affinity predictions were however difficult to rigorously assess because of the narrow range of experimental binding affinities. Other computational chemistry blinded trials exist, such as the D3R Grand Challenges, and can serve to supplement the SAMPL series [22]. This SAMPL7 challenge edition focused on fragment screening, binding pose prediction and follow-up generation from a database against a novel and pharmacology relevant bromodomain target, PHIP2.

PHIP was first found to interact with the Pleckstrin homology (PH) domain of the insulin receptor substrate-1 (IRS-1). IRS-1 is a tyrosine kinase involved in the signaling of many insulin-mediated processes such as mitogenesis and glucose transport [23]. Later, a larger PHIP isoform was localized in the nuclei of pancreatic beta-cells where it positively regulates cell growth and survival [24]. In addition, PHIP deficiency severely delays body growth and causes anaemia in young mice highlighting its important physiological function [25]. These findings associate PHIP to the insulin signalling pathway, therefore implicating this protein in tumorigenesis. Studies later confirmed that an increased PHIP copy number positively regulates metastasis in melanoma tumors that lack mutations in the 3 most frequently occurring oncogenic genes [26]. This opened a new avenue for specific therapies targeting BRAF-negative melanomas that account for a significant amount of all human melanomas and lack effective treatments. This strategy received further support when a study showed that the suppression of PHIP inhibits “driver-negative” melanoma, breast and lung tumor proliferation and invasion [27]. PHIP also enhances tumor cell mobility in glioblastoma cancer cells by acting on the focal adhesion complex [28], which is an important regulator of the actin cytoskeleton organization and dynamics. However, the precise molecular and structural mechanisms by which the multi-domain protein operates remain obscure despite the accumulating body of evidence that highlights its significant role in lethal cancers.

**Fig. 1:**
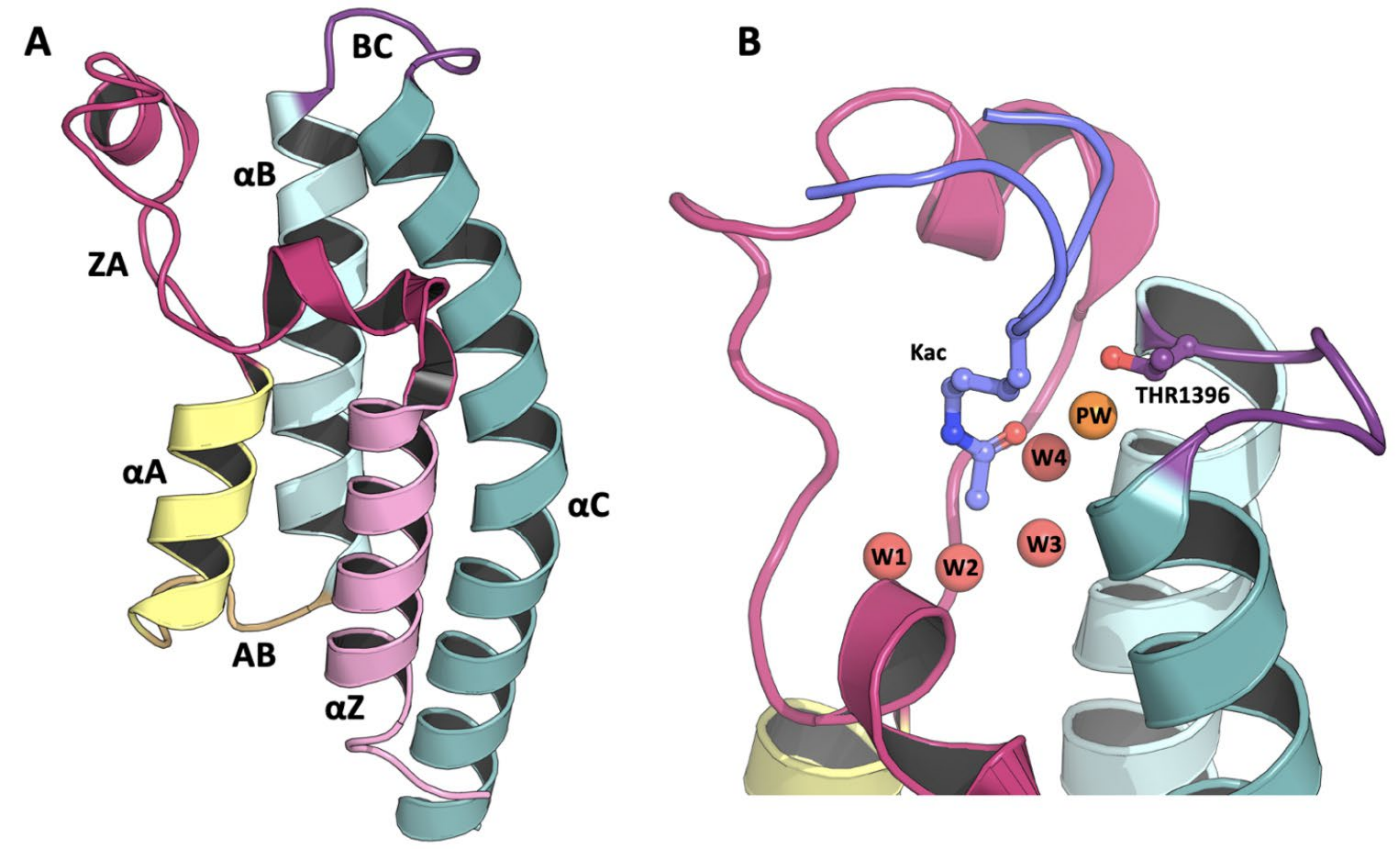
**Molecular structure of a typical bromodomain fold and PHIP2 binding site.** The left panel (A) shows the α-helical bundle fold (PDB-ID: 5RJI). The αZ, αA, αB and αC indicate the Z, A, B and C alpha helices, in pink, yellow, cyan and teal, respectively. ZA, AB and BC show the connecting loops of same name, in fuchsia, orange and purple, respectively. The right panel (B) shows the PHIP2 binding site in complex with H4K5acK8ac-like peptide (PDB-ID: 7BBP). The conserved bromodomain 4 water-network is showed in red spheres with the additional PHIP2 water in orange. The atypical threonine 1396 and acetylated lysine (Kac) are displayed in purple and lilac sticks, respectively

Overall, PHIP is a versatile protein that has multiple functions and subcellular locations. It is composed of 8 WD40 repeats and two bromodomains; PHIP1 and PHIP2. WD40 repeat domains are involved in a wide variety of cellular processes through molecular recognition events such as protein-protein or protein-DNA interactions [29]. Bromodomains are also part of larger multidomain proteins which are normally involved in transcriptional regulation or chromatin remodelling. They have a conserved α-helical bundle fold consisting of 4 α-helices namely, αZ, αA, αB, αC which are connected via more flexible loops named the ZA, AB and BC loops (Fig. 1).

Interestingly, bromodomains have a conserved network of four water molecules that sits at the core of the fold and facilitates the binding of an acetylated lysine (Kac) (Fig. 1B). These post-translationally modified amino acids are generally found on histone tails where they act as epigenetic markers. Thus, bromodomains act as specific “readers” where Kac is the central element of more complex peptide interactions where neighboring residues can also harbor other post-translational modifications such as methylation or phosphorylation. Through this interaction, bromodomains recruit other factors necessary for cellular function. From a pathological perspective, bromodomain-containing proteins are involved in many types of cancers. However, the role of the well-defined Kac binding site as a key mediator of protein-protein interactions defines them as attractive drug targets. An abundance of bromodomain inhibitors has been reported with several ongoing clinical trials [30]. In addition, chemical probe development offers an alternative to laborious laboratory work, such as engineered animal models, required to understand the cellular function of bromodomain-containing proteins.

Thus, a probe molecule that is specific to PHIP could open the way towards novel and broad-based chemotherapy against non-targetable tumors and/or facilitate the understanding of the biology behind these cancers. Computational, biochemical and proteomic data have indicated that the second bromodomain of PHIP (PHIP2) binds to the acetylated lysine 91 on histone 4 (H4K91ac) confirming the view that PHIP2 operates as an histone reader in the context of epigenetics [27].

PHIP2 is a member of the third bromodomain family and has an atypical Kac binding site because the highly conserved asparagine between the αB helix and the BC-loop is in this case a threonine (Fig. 1B). Such amino acid substitution is only observed in about 21% of known human bromodomains [31]. The substitution to threonine results in a less bulky side chain, therefore making space to accommodate an additional water molecule named the PHIP2 water (Fig. 1B).

In what follows, we first describe our analysis of the PHIP2 and the results of fragment screening itself before we then discuss how we set up the three stages of this particular SAMPL challenge; Stage 1 – discrimination of binders from non-binders, Stage 2 - Prediction of binding poses given known binders, and Stage 3 – Suggestions of follow-up molecules that might improve affinity.

## Methods

### Protein expression, purification, crystallization and X-ray screening

BL21 cells containing a pNIC28-Bsa4 vector coding for PHIP2 were taken from a glycerol stock (kindly provided by Dr. Tobias Krojer). 2 mL of Luria Broth pre-culture with 50 µM kanamycin were inoculated into 1 L Terrific Broth media with 2% glycerol (v/v), 0.01% (v/v) of 10% (v/v) sigma Antifoam 204 in ethanol, 50 µM FeCl_3_, 20µM CaCl_2_, 10 µM MnCl_2_, 10 µM ZnSO_4_ and 2 µM of CoCl_2_, CuCl_2_, NiCl_2_, Na_2_MoO_4_, Na_2_SeO_3_ and H_3_BO_3_, 2 mM CaCl_2_, 25 mM ammonium sulfate, 2.77mM glucose and 50 µM kanamycin. The cultures were grown for 6 h at 37 °C at 250 rpm. PHIP2 expression was induced overnight at 18 °C with 0.1 mM IPTG.

Cultures were centrifuged at 4000 g for 30 min at 4 °C. Pellets were resuspended in lysis buffer (10mM HEPES, 500mM NaCl, 5% Glycerol, 0.5mM TCEP, 0.5 mg/mL Lysozyme, 1 g/mL Benzonase, pH 7.5). The solution was vortexed and left at room temperature for 30 min before. 2% triton-X and 20 mM imidazole finale concentrations were added to the mixture before being centrifuged at 4000 g for 30 min at 4 °C. The supernatant was applied onto a 1 mL His GraviTrap columns (GE healthcare) fitted with a LabMate extender. The columns were washed twice with wash buffer (10 mM HEPES, 500 mM NaCl, 5% Glycerol, 0.5 mM TCEP, 20 mM Imidazole, pH 7.5). The columns were slotted PD10 columns fitted with LabMate extenders. The proteins were eluted by applying 2.5 mL of elution buffer (10 mM HEPES, 500 mM NaCl, 5% glycerol (v/v), 0.5 mM TCEP, 500 mM Imidazole, pH 7.5) onto each GraviTrap column. 3.5 mL of wash buffer was applied onto each PD10 column and elutions were collected. 1 OD280 unit of TEV protease per PHIP2 10 OD280 units was added to the elutions and incubated at 4 °C. The solutions were run back over His GraviTrap columns as mentioned above. The fractions were concentrated by 20-fold and applied onto a Yarra Sect. 2000 pre-equilibrated with wash buffer. The fractions containing the protein were collected using either a biorad C-9 or a Cytiva ALIAS. The fractions were concentrated to about 15 mg/mL of protein and flash-frozen in liquid nitrogen.

PHIP2 was crystallized in space group C2 at 4 °C by vapour diffusion in 230 nL sitting drops, by mixing 100 nL protein in wash buffer with 100 nL reservoir buffer (20% PEG8000 and 40 mM potassium phosphate) and 30 nL seeds of the same composition than reservoir. The final pH was measured to be ~ 5.6.

Crystals suitable for fragment screening were located in the plates with TexRank [32]. These were soaked with 20mM final concentration of each fragment and 20%(v/v) ethylene glycol using an ECHO acoustic liquid handler dispenser. The crystals were incubated for 2 h at 5 °C and harvested with a SHIFTER before being plunged into liquid nitrogen and shot at the i04-1 beamline located at the Diamond Light Source (Harwell, UK). The XChemXplorer [33] was used for crystallographic workflow management and paralleling. Molecular replacements and initial refinements were performed with DIMPLE [34]. Pandda [6] was used to identify low occupancy binding events. Ligands were fitted in Coot [35] and the structures refined with Buster [36] and deposited on the protein data bank (PDB) with deposition ID: G 1,002,162.

## Metrics for calculations

The molecular descriptors (molecular weight, logP, topological surface area, Tanimoto coefficients, H-bond donor, H-bond acceptors, rotatable bonds and rings) were computed with RDKit (v2020.03.2.00) [37].

Sensitivity, specificity, and balanced accuracy were calculated with scikit-learn (v0.22.1) where scores were computed against the experimental ground truth array where crystallographic fragment hits and non-hits were considered as definitive. The 40 fragments that did not lead to any usable diffraction data were not taken into consideration. The smiles strings of the fragments that did not results in usable electron densities can be found in **SI** Fig. 1. This resulted in 52 positives and 707 negatives, across all sites. 47 positives and 712 negative were identified at the Kac binding site.

Submissions confidence intervals were estimated by resampling submission arrays using bootstrapping with a percentile of 0.95 and 10,000 samples. In order to calculate root mean square deviations (RMSDs), the docked fragments were merged with their corresponding receptor PDB files and their α-carbons were then aligned onto the ones of the experimental structure with MDAnalysis [38, 39], therefore shifting the fragment along with the transformation. The docked vs. experimental fragment RMSDs were then calculated with spyrmsd (v0.3.4) [40], which takes into account molecular symmetry.

All the experimental data, participant submissions, analysis and codes can be found here: https://github.com/samplchallenges/SAMPL7/tree/master/protein_ligand.

## Fragment network

Datasets with candidate molecules were generated by querying data from the fragment network [41], which is graph database that allows a user to efficiently search chemical space around a compound of interest and has been reimplemented at the Diamond Light Source using Python and RDKit [37]. The source data comprises a subset of ~ 40 million molecules from the Enamine REAL database [42] from 2018 that had similarity to the DSI poised library [31] used in fragment screening at Diamond as well as ~ 7.5 million molecules from the Molport “All stock compounds” data set. The fragment network data was available in a Neo4j database queried using the Cypher language for each of the 52 source molecules. Results for each of the 52 queries were aggregated and written in SMILES format along with the supplier identifiers. Both chiral and achiral molecules are present. The aggregated queries containing follow-up compounds can be found at https://zenodo.org/record/3576140#YSVJhXVKjmx.

## Results

### Crystallographic fragment screening of PHIP2 reveals novel binders

PHIP2 was crystallized in a C2 space group that diffracts to a resolution of approximately 1.2 Å. This crystal form is easily reproducible making it ideal for an XChem screen [3]. The DSI-poised [31] and FragLites [43] libraries were screened against the C2 crystals. The former library is composed of 768 fragments and was designed to ease follow-up chemistry. FragLites is composed of 31 halogenated fragments, all of which have a paired H-bond donor/acceptor motif to probe minimal interaction doublet in the binding site while the halogen atoms assist electron density fitting. Out of these 799 fragments, no binding was observed for 707 of them despite the acquisition of adequate diffraction data sets. To minimize to the number of false negatives, each Fraglites fragment was screened in duplicate. The remaining fragments were re-screened if the diffraction dataset did not display the expected C2 space group or had resolution lower or equal to 2 Å or if the *R*_*crys*t_ and *R*_*fre*e_ values were lower than 0.23 and 0.25, respectively. This led to the re-soaking of 202 fragments that resulted in the identification of 10 additional hits including 8 Kac binders. No data was collected for 1 FragLites (F12) and 39 other fragments solutions led to consistently damaged crystals (despite repeated soaking) resulting in the absence of diffraction data for these molecules (**SI** Fig. 1**)**. The exact cause(s) of such degradation remain, in this case, unclear and could be diverse but may be due to relative crystal tolerance to individual ligands at the soaking concentration or defective ligand stock solutions.

**Fig. 2:**
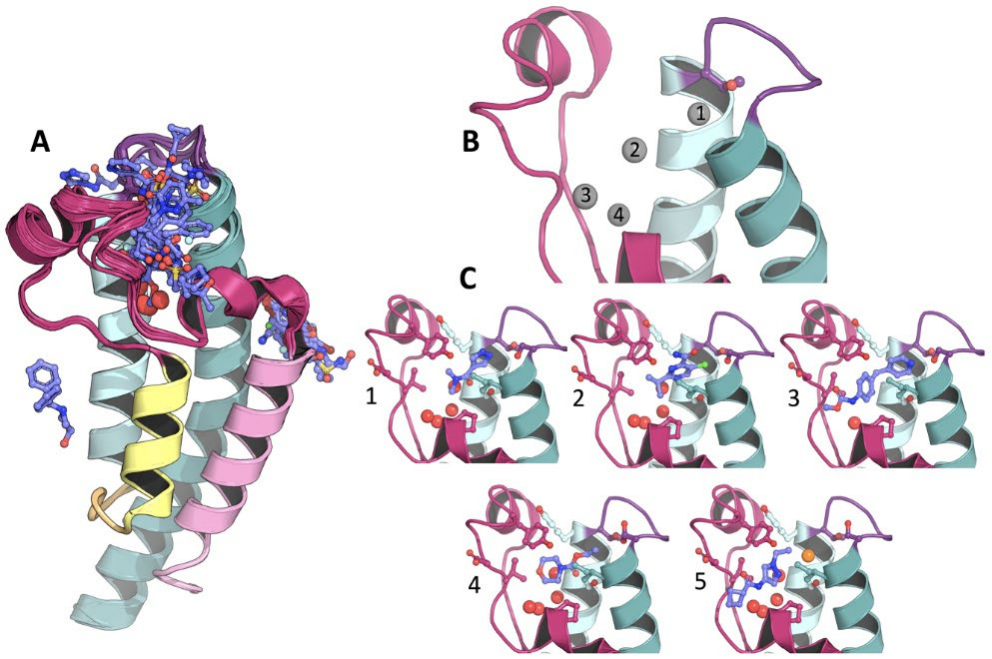
**Overview of the fragment hits against the C2 crystal form.** (A) Overlay of all structures resulting from the crystallographic high-throughput screening. The Kac binding is the most populated site. The other sites can be seen on the right and left of the Kac binding site. An additional fragment hit is located behind the purple BC-loop. (B) illustrates the 4 fragment binding subsites with the B1, B2, B3 and B4 denotating the BC-interface, central void, ZA-channel and the water cavity, respectively, as grey spheres. (C) shows selected fragment binding poses to illustrate the diverse types of interactions and binding regions identified from the results. C1 shows F421 (PDB: 5RK9) that forms an H-bond with SER1392 at the BC-interface. C2 shows F126 (PDB: 5RKE) that forms a halogen bond with THR1396 and SER1401 at the BC-interface. C3 shows F558 (PDB: 5RJV) which has 2 aromatic 6 membered rings that both form perpendicular pi-stacking with TYR1350 and TYR1395. C4 shows F393 (PDB: 5RK7) which has a morpholine moiety that fills the central void. Finally, C5 shows F368 (PDB: 5RKN) which forms H-bonds on the ZA-channel with backbone nitrogen and oxygen of ASP1346 and PRO1350, respectively

**Fig. 3:**
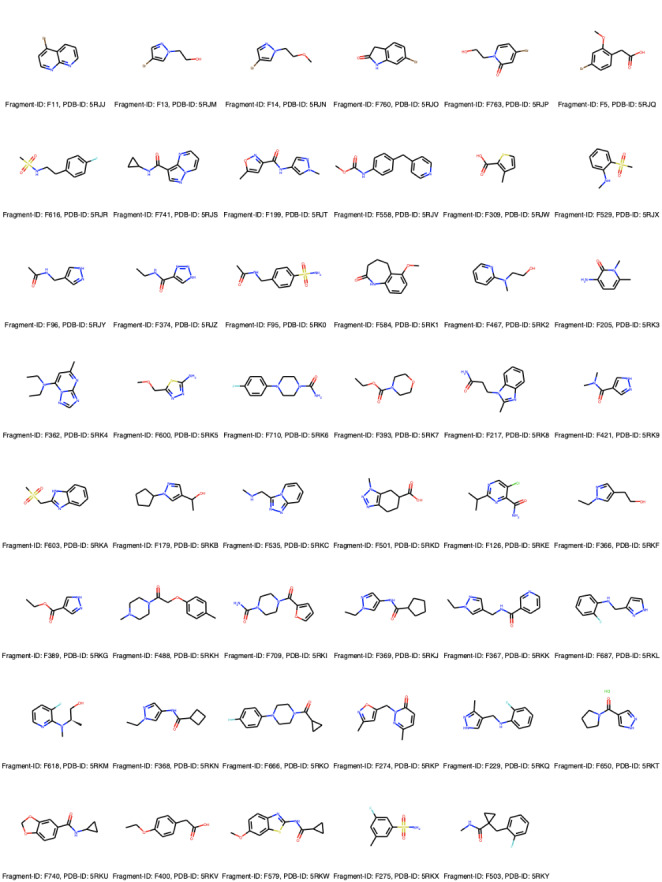
**The 47 Fragments identified in the Kac binding pocket of PHIP2.** The fragment IDs and corresponding PDB accession numbers are showed, below each 2D molecular representation on the left and right, respectively. The Smiles, PDB codes and fragment IDs are available on the GitHub page of the challenge: https://github.com/samplchallenges/SAMPL7/tree/master/protein_ligand/experimental-data/stage-2 under pdbs_overview.csv

52 hits were identified across 4 sites (Fig. 2) therefore achieving a global hit rate of 6.51%. 47 fragment hits are bound to the pharmacology relevant Kac binding site (Fig. 2 A) and are summarized in **Fig **3. 7 hits were resolved at a small, solvent-exposed cavity located between helices C and Z and 4 out of these also bind to the Kac binding site. 2 additional hits were found, 1 behind the BC-loop and 1 in between the helices A and B (Fig. 2). The fragments binding away from the main Kac binding site are largely solvent exposed and contact other protein molecules in the lattice and hence may be artifacts of crystal contacts – that is, representing binding which would not occur if the protein were in solution. Thus, only the Kac binding site hits were considered going forward.

Chemotypic analysis of the fragments bound to the Kac site suggests they tend to be smaller in molecular weight and more hydrophobic than the library average and the majority of them have 1 and 3 H-bond donors and acceptors, respectively. Overall, the hits (and library compounds) have relatively low chemical similarity with an average Tanimoto coefficient of about 0.23. A more detailed chemotype analysis is summarized in **SI** Fig. 2.

Visual inspection of fragment binding to the Kac binding site suggests this pocket can be considered as 4 sub-sites (Fig. 2B). These were named: (i) The BC interface, which includes interactions with αB, αC, and the BC-loop (ii) the water cavity, which is defined by the location of the 4 water-molecule network (iii) the ZA channel, which is the part of the ZA loop that forms a semi-circle (iv) the central void which lies at the center of the 3 other sub-cavities. The subsites and exemplar fragments are displayed of Fig. 2B C, respectively. The screen probed the Kac binding site very well with fragments contacting almost all side chains composing its surface (Fig. 2 A). The most frequently occurring H-bond forming protein groups were the side chains of Ser1392, Thr1396 and Ser1401, all of which are located at the BC-interface as well as the backbone nitrogen of Asp1346 and the backbone oxygen of Pro1340, which are located on the ZA channel. Halogen bonds show the same pattern of interaction around the BC-loop but penetrate deeper into the protein to interact with multiple side chains simultaneously. Tyr1350 seals the top of the central void, whilst Tyr1395 is located on the BC-interface. These side chains are positioned in such a way that fragments can only form perpendicular pi-stacking. Other hydrophobic groups that frequently contact the fragments are Val1345, Ile1403, which are in the central void. The PHIP2 water appears to be easily displaceable as all fragments that interact at the BC interface displace it (Fig. 2 C).

**Fig. 4:**
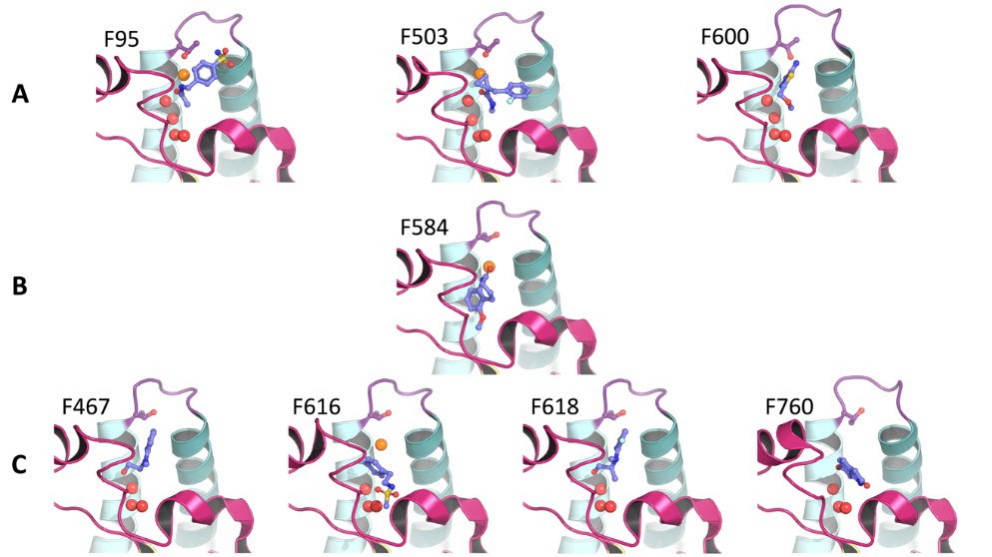
**Special Kac fragment binder cases.** (A) shows fragment poses that induce re-arrangement of Thr1396 into a peptide-bound like conformation, which is paired with a change of the BC-loop conformation. (B) shows the deep binding pose of F584, such that it displaces the whole water network. (C) shows four fragments that displace W4 only. The PDB codes for each fragment are available in Fig 3

Overall, the C2 crystal form appears to be rigid with few fragments inducing protein motions. F760 induces the largest protein motions by relaxing the ZA- and BC-loops away from the binding site and bringing Tyr1395 closer to the core. Interestingly, fragments F95, F503 and F600 cause the re-arrangements of Thr1396 into a peptide-bound conformation (Fig. 1B) where the side chain hydroxyl groups point towards the inside of the binding site. In F95 and F503, this is paired with the formation of a water bridge between the fragment and Thr1396 whilst F600 directly contacts this side chain (Fig. 4). In addition, the 2 former fragments rotate Ile1403 away from the binding site in a parallel orientation with αC.

5 fragments disrupt the 4-water network to different degrees (Fig. 4B-C). F584 is the most remarkable one by displacing all 4 water molecules to locate itself deep in the binding cavity where its benzene moiety interacts with Tyr1353 and its 7-membered ring fills the hydrophobic space of the central void. F467, F616, F618 and F760 all displace the fourth water of the network. This water was predicted to be the least stable of the network by Grand Canonical Monte Carlo analysis [44].

## **SAMPL – Stage 1. Discrimination of binders from non-binders at specific sites**

The aim of the first stage was to discriminate binders from non-binders identified at the Kac binding site from screening presented above. The participants were provided with the isomeric SMILES strings of the 799 fragments composing the mixed library as a .csv file. In addition, they were also provided with a PHIP2 apo-structure in the aforementioned C2 space group. The location of the Kac binding site was also indicated by the positioning of a dummy noble gas atom positioned in the PDB file. The experimental screening protocol was also supplied so that the participants would have all the information available to computationally reproduce the crystallographic results. The experimental information provided included the crystallization conditions, the pH, and the final fragment soak concentration. The detailed protocol was given so that the participant could take into account the chemical conditions surrounding the experiment. A submission template was also provided which participants were required to use to submit predictions, and entrants were given 1 month to submit entries. The participants were asked to categorise each fragment listed with Boolean values: True and False for binders and non-binder, respectively. We did not ask for any ranking, scoring, or confidence metric to be reported. Submissions were requested for all four binding sites seen in the screen. However, most participants only submitted solutions for the main Kac sites and thus we do not discuss these peripheral sites any further. Table 1 provides a summary of submissions, which we expand on in detail via the individual submission identification numbers (SIDs).

**Table 1:** **Overview of Stage 1 virtual screening submissions.** 9 sets of predictions were submitted for Stage 1. The first, second, third and fourth columns correspond to the submission identification number (SID), the affiliation of the participants, the method’s name and the list of software used. The last column shows the ratio of predicted binder over predicted non-binders

SID	Participant Affiliation	Method Name	Method category	Software used	Non-Binders to binders ratio
38	Akiyama Lab, Department of Computer Science, Tokyo Institute of Technology	AutodockVina-VirtualScreening	Docking	Autodock Vina 1.1.2, MOE 2019.0102, Openbabel 3.0.0, AutodockTools 1.5.6	0.001
55	Not provided	dock_score	Docking, (MD)	OpenEye docking toolkit v1.1	7.528
33	SAMPL7	Null	Random choice	Random predictor	9.397
56	The University of Tokyo, Japan	Template docking and similarity search	Docking, Ligand-based	Molegro Virtual Docker (7.0.0), Schrödinger suite (2019-3), PubChem (current)	10.859
52	Institut de Chimie des Substances Naturelles, CNRS, Gif-sur-Yvette, France	docking-S1-G2-chemscore-top5perc	Docking	Schrodinger LigPrep v48012, CACTVS Chemoinformatics Toolkit V3.4.6.26, CORINA v4.2.0, CCDC GOLD v5.7.1 (CSDS-2019-1)	17.975
35	University of Oxford, UK	Molecular Docking with MM-GBSA Scoring	Docking, MD	OpenBabel v3.0, UCSF Chimera v1.12, PDB2PQR v2.1, SPORES v1.3, PLANTS v1.2, DOCK v6.8, AmberTools v2019,	20.686
37	Universitat Pompeu Fabra, Spain	ECFP4rdkit-RF	Ligand-based, ML	MOE 2016.08, python 3.6.9, rdkit 2019.09.1.0, scikit-learn 0.21.3,	68.000
43	Institute of Systems Biomedicine, Peking University	Deep Learning; k-deep; docking	Docking, ML	Python, RDKIT,	68.000
44	University of Barcelona, Spain	DUck-aided Virtual Screening (DaViS)	Docking, MD	rDock 2013.1, Amber16, AmberTools16, MDmix 0.2.0, Gromacs version 2018.1, LigPrep version 46,013, MOE 2019.0102, Prime version 5.6 (r012)	25.172

SID 38 used a typical workflow, whereby ligand conformations and protein protonation states were generated by MOE, which was followed by docking with AutoDock Vina [45]. A threshold of ≤ 4.0 kcal/mol for the best scoring pose was used to categorize a fragment as a binder

SID 55 used OpenEye Omega Toolkit [46] for conformer generation and docking, respectively with docks being scored with Chemgauss4 scoring function. The participants behind SID 55 did not provide us with their names, affiliations and threshold employed to discriminate binders from non-binder

SID 33 corresponds to a negative control random submission where compounds were selected randomly of compounds were selected as binders using an *in-house* python script.

SID 56 employed two separated protocols. The fragment-hits from [31] were listed and used in a PubChem 3D and fingerprints similarity searches and hits that were screened fragments were considered as binders. Then, the remaining fragments were protonated at crystallographic pH then template docking was employed against 2 fragment-bound PDBs in a different (P21212) symmetry group. The participants mentioned that they considered docking scores, similarity scores and ligand efficiency when defining binders and non-binders without further explanations.

SID 52 first built a validation set of 1499 active compounds from assay and crystallographic data which includes 45 fragments belonging to the provided library. 3D conformers for each validation active were generated with Corina [47] and protonated at physiological pH with Schrodinger’s Maestro tool. Gold was used with 4 scoring function (GoldScore, ChemScore, ASP and ChemPLP) to dock the 1499 validation actives onto 3 distinct fragment conformers identified from 8 fragment bound PDB structures [48]. They then looked at the ranking of the 45 library validation active fragments as well as RMSDs to all 8 fragment-bound PDBs. ChemScore and the second of the 3 fragment conformer structures yielded the best scores. They used those parameters to subsequently dock the 799-fragment library and defined the top 5% as binders.

SID 35 kept 5 water molecules during the preparations step with DockPrep tool [49]. Protein and ligand were protonated with PDB2PQR and OpenBabel [50], respectively before performing docking with PLANTS [51] using the ChemPLP as scoring function. The poses were further scored using DOCK6 [49], which performs minimization, MD simulation and generalized Born/surface area (GB/SA) continuum model scoring in Amber.

SID 37 was the only submission that did not employ docking at any stage of the predictions. Instead, they built a random forest classifier based on data from publications, ChEMBL and the PDB. The training set was composed of structural data related to PHIP2, directly, obtained from the DSI poised library [31], where they identified a total of 10 and 448 binders and non-binders, respectively. An additional 1214 structures were obtained from the PDB by searching for other bromodomain family members, the associated ligands were extracted and classified as binders. 70,000 additional molecules were retrieved from ChEMBL and classified as binders based on activity measurements. Crystallographic and ChEMBL ligands were protonated at pH 6 and 7, respectively. Model training was performed against chemical descriptors and Morgan fingerprints. Finally, they tested their machine-learning model against the provided SMILES and molecules were predicted to be binders if their probability score was higher than 0.5.

SID 43 did not provide us with their protocol describing the method employed for predictions despite multiple requests. A combination of machine learning and docking was seemingly employed.

SID 44 employed different MD-based methods and docking in their workflow. The stability of the water network was, first, assessed with MDmix [52] and the participants concluded only water-4 to be easily displaceable. Classical MD simulations were then employed against the provided Apo state and ligand bound structures identified from [31] to evaluate protein motions. They observed that the ZA-loop is the most flexible region by visiting opened, semi-opened and closed states and that the presence of ligands stabilizes that protein in an opened state. Thus, another structure that displays a more open state of the ZA-loop was selected for further processing instead of the provided Apo state. They then used rDock [53] with pharmacophore restraints to generate poses. 3 different protocols, which used different sets of restraints and water molecules, were applied onto the provided smiles after tautomers and protomers enumeration at pH 5.6 +/- 1. For each fragment, the resulting poses were first clustered and then finally selected based on the protein-ligand interaction score. The docking poses resulting from each 3 protocols were evaluated with a Dynamic Undocking (Duck) [54] like procedure which extracts the ligand and nearby binding-site environment, performs steered-MD and measures the work done to assess the strength of the interaction. A threshold was used to categorize binders from non-binders with binders scoring lower than 2 kcal/ mol. Further refinement was applied to the predicted binders list which excluded the ones displaying unstable docking pose through the Duck MD windows.

To assess the performance of each submission we computed the sensitivity and specificity, which are the True Positive and True Negative rates, respectively. The classified predictions were compared to the ground truth values defined by the outcome of our crystallographic fragment screening experiment. Here we defined an experimental positive when an interpretable electron density in which is fragment can be fitted is observed and a negative where such signal is absent. It is difficult if not impossible to discriminate non-binding events that are caused by structural (i.e. binding allowed by receptor’s conformation(s) and/ or energetics) or chemical factors (such as crystal conditions). Negatives therefore correspond to experimental negatives, irrespectively of their underlying nature. Since our dataset is largely populated of non-binders, we used balanced accuracy to evaluate the results. The balanced accuracy is the simple arithmetic average of these 2 metrics and is equivalent to the area under the ROC curve. A score of lower, equal, or higher than 0.5 indicates a worse than, equal to or better than random prediction.

**Fig. 5:**
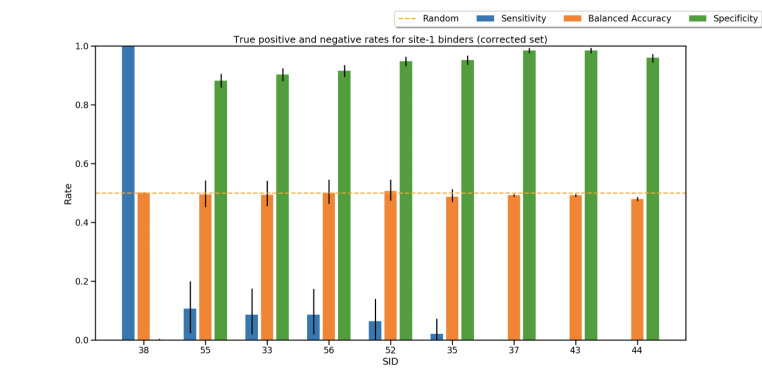
**Performance summary of submission to Stage 1 of the challenge.** The Sensitivity, Balanced accuracy and Specificity rates for each submission are shown in blue, orange and green, respectively. The error bars associated which each metric was estimated via bootstrapping of the data and the orange horizontal line shows a random perfection based on balanced accuracy values

This stage turned out to be extremely challenging with all submissions performing approximately randomly with an average balanced accuracy across all submissions of 0.494 ± 0.007 (Fig. 5). Overall, all methods, but SID 38, tended to correctly classify a large majority of the fragments as non-binders as indicated by their high specificity rates. However, these failed to identify most binders with submissions 37, 43 and 44 being unable to identify a single binding event and submissions 55, 56, 52 and 35 only correctly identifying 5 (F95, F199, F558, F579, F740), 4 (F13, F14, F96, F488), 3 (F275, F529, F760) and 1 (F362) binding events out of 47, respectively (**Fig **3). SID 38, however, showed an opposite outcome where all fragments were categorized as binders therefore leading to a Sensitivity rate of almost 1.

## SAMPL - stage 2. Prediction of binding poses for known fragment binders

The second part of the challenge built upon the first stage. The objective was to correctly predict the 3D binding pose for the 47 protein-fragment complexes resolved by X-ray crystallography at the pharmacologically relevant Kac binding site. The participants were provided with the same C2 apo state structure that was supplied in Stage 1. In addition, the SMILES strings of the binders were provided in a .csv file. It was also specified that the compounds were purchased as racemic mixtures and that higher affinity conformers should be revealed in the electron density or that both stereoisomers could bind, thus resulting in an average electron density [55]. A submission template was also provided to aid analysis and entrants were given about 2 weeks to submit entries. Participants were tasked to submit at least one and no more than 5 poses for each of the 47 fragments. The poses were also asked to be ranked (from best to worst) if more than one was submitted. Finally, participants were strongly reminded that predictions should be done considering the C2 space group as crystallographic screening against different crystal forms often leads to differential fragment-hit identification [56], which may be due to due to solution- and solid-state effects such as pH and crystal packing. We knew that this is also true for PHIP2 as a previous screen against a P21212 form [31] revealed different fragment binders than against our C2 form. We, however, decided to hide that information to strengthen the blinded aspect of the challenge and evaluate how the participant select their system. Only 5 submissions were received for this stage of the challenge for publication, summarized in Table 2.

**Table 2:** **Overview of the Stage 2: Binding pose prediction submissions.** 5 sets of predictions were submitted for Stage 2. The first, second, third and fourth columns correspond to the submission identification number (SID), the affiliation of the participants, the method’s name and the list of software used

SID	Participant Affiliation	Method Name	Method category	Software used
77	Molecular Modeling Section Lab (Prof. Stefano Moro) University of Padova, Italy	HT-SuMD	Other, (MD)	MOE 2019.01 Acemd3 VMD Python 3.6 scikit-learn 0.21.3 AmberTools 2016
75	Acellera	rDock-rDeep	Docking, ML	rdkit 2018.03.4 HTMD Playmolecule proteinPrepare rDock rDeep v0
64	Not provided	2d feature model	Docking	Smina
79	The University of Tokyo, Japan SHaLX Inc.	Template docking	Docking	Molegro Virtual Docker (7.0.0)
80	Institut de Chimie des Substances Naturelles, CNRS, Gif-sur-Yvette, France	ranking_stage2	Docking	Schrodinger LigPrep v48012 CACTVS Chemoinformatics Toolkit V3.4.6.26 CORINA v4.2.0 CCDC GOLD v5.7.1 (CSDS-2019-1)

All methods but one used some form of docking for binding pose prediction. Only SID 77 was totally agnostic of docking. Instead, they used Supervised Molecular Dynamic Simulations (SuMD) [57]. SuMD is a relatively novel MD-based method that aims to sample binding and unbinding paths between a ligand-receptor pair. The algorithm positions the ligand in an MD box, monitors the translation of the ligand towards the binding site and selects frames that are closer to the target binding site. Once the ligand is sufficiently close to the binding site (around 5 Å), the engine switches to a classical unbiased molecular dynamic simulation of the protein-ligand complex. Finally, the final unbiased production runs are saved, analysed, and scored. Here, triplicate SuMD runs were carried out and the relevant conformations, for each fragment, were extracted from production simulations using a DBSCAN clustering. The different clusters were ranked by a consensus approach that accounts for the cluster sizes, MMGBSA energies and MOE-Hyd estimation of the hydrophobic contributions to binding. Finally, the centroid structure for the top 5 scoring clusters were selected.

SID 75 kept structural water molecules and prepared their system at pH 6.0 in playmolecules.com [58]. 100 poses per fragment were generated with rDock [53]. which were rescored with a seemingly in-house, convolutional neural network function and the top 5 non-redundant poses were submitted.

SID 64 used a simple docking protocol where they AutockVina [45] and docked the fragments into Kac binding site of the provided apo structure. The participants gave little additional information about their protocol.

SID 79 selected 3 ligand-bound PHIP2 PDB structures: 3MB3, 5ENF and 5ENI. The provided binders were classified by template matching which resulted in 3MB3, 5ENF and 5ENI being associated with 19, 15 and 12 binders, respectively. Molegro Virtual Docker [59] was used for docking of the binders into the receptors. The provided apo structure was only used in one case after alignment of 3MB3.

SID 80 used a similar method to their Stage 1 submission. The same validation set of 1499 compounds which contained 45 actives was used. The systems were prepared as mentioned above and the fragments were each docked onto 3 different ligand conformations with 50 poses generated for each. Again, ChemScore on the second conformation performed best. The provided fragment-hits were prepared and docked using the workflow described above and the top scoring pose submitted.

**Fig. 6:**
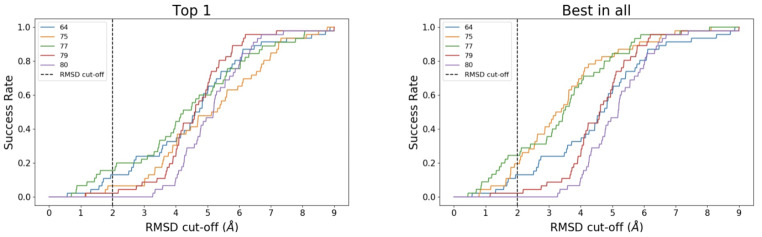
**Success rate variation as a function of RMSD cut-off value.** The left (Top 1) and right (Best in all) panels show the variation in success rate for the best scoring and lowest RMSD poses

The performance of each submission was assessed by calculating the fragment root mean square deviation (RMSD) between the docked and the crystallographic poses. The top scoring and lowest RMSD poses amongst submitted poses for a given fragment, were recorded for success rate evaluation. Calculation of the success rate as a function of RMSD shows how a particular submission performs as we change the RMSD cut-off defining success rate. However, in line with many studies in the literature, a RMSD cut-off of ≤ 2Å was chosen to categorized docking pose predictions as successful, while all docks beyond this value were categorized unsuccessful (Fig. 6). It should be remembered that RMSD values beyond 2 Å are quite poor docking poses. The top 1 correspond to the top ranked pose and best in all to the most accurately predicted pose.

**Fig. 7:**
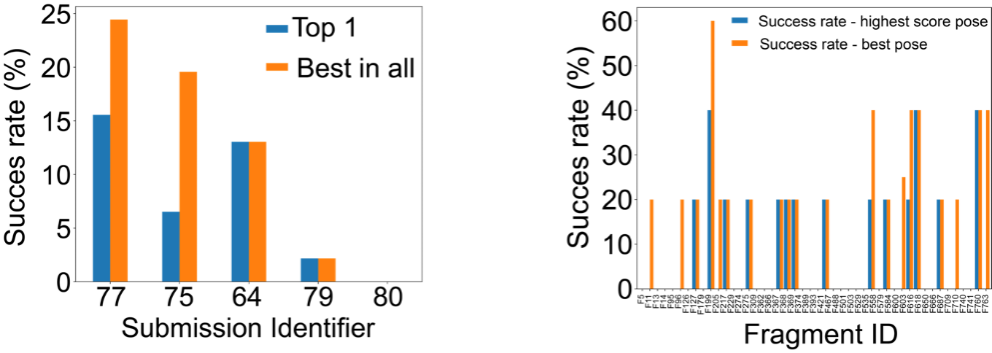
**Submission’s success rate assuming and RMSD cut-off of 2 Å.** (A) compares overall success rate between SIDs. SID 77 is the best performer, but even there the success rate is still low. (B) Success rate per fragment ID. Only 3 fragments are predicted correctly by more than one group

There was no difference in success rates between the Top 1 and Best in All for SIDs 64, 79 and 80 as these participants only submitted one pose per fragment. This resulted in a better performance of methods 75 and 77 when the lowest RMSD pose is considered over the best scoring pose (Fig. 7).

Overall, the performance of the methods was disappointing (Fig. 7). The method that was the most capable to reproduce binding poses at an RMSD of 2 Å or lower was SID 77, which performed best for both ranking methods. SID 77 predicted correctly 11 and 7 fragment poses out of 45 for the top 1 and top 5, respectively. SID 77 treated the whole system dynamically, which resulted in binding site conformations different to the one observed in the provided C2 crystal form. However, this did not prevent them correctly identifying the binding pose of 16% and 24% of the binders for the Top 1 and Best in all, respectively. The success of submissions 75 and 77 was significantly enhanced when looking at the Best in all over the Top 1 which was 9 and 13% higher, respectively. Thus, the SID 75 success experienced the most dramatic drop between best in all and top 1. For the top 5, SIDs 75 and 77, however performed better than the others that did not submit 5 poses implying that, in this case, the best scoring pose is not necessary the one with the lower RMSD. Submissions 79 and 80 performed relatively poorly with success rates of 2% and 0%, respectively. These poor results might reflect the fact that the provided C2 apo state was not used in the dockings. Instead, other, ligand-bound structures were used as receptors, which have different binding site conformations. Evidently, the scoring/rescoring scheme used in submissions 79 and 80 were targeted toward different and static binding site states that prevented the identification of the correct binding poses. Contrarily, submissions 64 and 75 did employed the provided C2 structures which led to an improvement in the results when compared to submissions 79 and 80.

When looking at the success rates for individual fragments (Fig. 7) no clear correlation could be robustly established given the small number of submissions, which translated into an even smaller number of predictions per ligand. Qualitative observations could, however, be made. F205 was the fragment predicted with the highest success rate for the Top 1. F205 was predicted correctly in 3 and 2 submissions out of 5 for the top 1 and top 5, respectively. This relatively polar and flat ligand fills the central void and makes an H-bond with Ser1392. F558, F616, F618, F760 and F763 were correctly predicted twice for the best in all. F558 one of the largest of the fragments that binds at 3 out of 4 subsites (the BC-interface, the central void and the ZA-channel) and fills most of the binding site volume. Interestingly, F616, F618 and F760 all displace the 4th water molecule from the water network, which highlights how correctly accounting for water molecules dynamics and/ or positioning can lead to better results in binding pose predictions for particular fragments. F763 has a similar chemotype and binding pose to F205. Overall, this stage appeared to be particularly challenging.

## SAMPL - stage 3. Selection of fragment follow-up molecules from a database

The goal of the third and final stage of the challenge was to enumerate fragment follow-up compounds from a provided database. These follow-ups should target the PHIP2 Kac binding site with higher potency than the original crystallographic fragment hits. The participants were provided with the co-crystal structures of the Round 2 fragment hits to support their selection process. They were also given a library of more than 40 M molecules, which was a combination of the Molport “AllStockCompounds” (dating from July 2019) and a subset of the Enamine Real© library [42]. The rationale behind the supply of this particular library was that the compounds could simply be purchased from either Enamine or Molport for follow-up experiments.

In addition, library subsets generated by the fragment network [42] were also provided in case participants preferred to work with smaller numbers of compounds. The fragment network provides a convenient way to identify similar compounds. Molecules are fragmented by the removal of substituents, rings and linkers and a graph database assembled with nodes corresponding to molecules and their sub-fragments and edges corresponding to the parent-child relationships between those nodes. The search algorithm requires an input molecule and 3 parameters: 1- the number of edges to traverse (hops) from the query molecule, 2- number of changes in heavy atom count (hac), 3- number of changes in ring atoms counts (rac). The fragment hits were used in queries with number of hops, changes in heavy atom count and number of changes in ring atoms counts ranging between 1 and 4, 3 and 5 and 1 and 2, respectively, with the resulting molecules typically corresponding to variants of the query with substituents, rings and linkers added and, deleted and/or replaced.

Participants were asked to suggest between 10 and 100 follow-up compounds from the provided library, along with a confidence score, for each follow-up, between 0 and 10, with the former and the latter meaning a low and high confidence in the follow-up binding and increasing potency, respectively. We also (re)emphasised that the experimental setup and binding assay (here crystallography) must be kept in mind when making predictions. The participants were encouraged to submit poses if using MD- or docking-based methods. The entrants were given 1 month to submit their follow-ups.

Initially, the fragment follow-up compound would have been purchased and experimentally validated against the C2 crystal form at the XChem by X-ray crystallography. The follow-ups and generation methods would have been examined by medicinal and computational chemists to assess whether the molecules were worthy of being bought. For these reasons, only one submission and no null models were allowed. A total of 50 to 100 compounds were expected to be purchased given our budget at the time of setting up stage 3. Unfortunately, the COVID-19 pandemic resulted in a diversion of funds before this follow-up could be done, and the challenge terminated at this point. Here, instead, we will critique the top 10 submitted follow-ups and comment on their value as likely molecules for purchase. Only 4 submissions were received in total, summarized in Table 3.

**Table 3:** **Overview of the Stage 3 follow-ups enumeration submissions.** 4 sets of predictions were submitted for stage 3. The first, second, third and fourth columns correspond to the submission identification number (SID), the affiliation of the participants, the method’s name and the list of software used

SID	Participant Affiliation	Method Name	Method category	Software used
82	Peking University	XGboost_gnina_Yu	Docking, ML	rdkit xgboost gnina
85	Acellera	SkeleDock-rDock	Docking	SkeleDock RDKit HTMD rDock
86	Institut de Chimie des Substances Naturelles, CNRS, Gif-sur-Yvette, France	ranking_stage3	Docking	Schrodinger LigPrep v48012 CACTVS Chemoinformatics Toolkit V3.4.6.26 CORINA v4.2.0 CCDC GOLD v5.7.1 (CSDS-2019-1)
87	The University of Tokyo, Japan SHaLX Inc.	Template filtering then template docking	Docking	RDKit (2018.09.1) Molegro Virtual Docker (7.0.0)

Overall, this stage was divided into 2 implicit subparts: database filtering and ligand selection. 3 submissions did indeed apply some form of filter whilst 1 did not. Then, all methods employed a docking protocol to score the ligands. Only 1 method employed an alternative to their usual docking/ scoring schemes and that was a machine-learning based approach. No submissions employed an MD based method (likely reflecting the time constraint). Only submissions 85 and 87 submitted docking poses despite our encouragement to do so. We had encouraged such submissions because we felt that plausible poses might provide human experts (those making predictions or those selecting compounds for experiments) with additional incentive to select compounds for follow up, and that implausible poses could lead such experts to rule compounds out.

SID 82 failed to provide a complete description of the method employed for follow-up identification. However, the method appears to have filtered the library using an Xboost classifier built as part of Stage 1. The database molecules were represented as MACC fingerprint and the classifier achieved a mean area under the curve of 0.73 with 10-fold cross validation. The top 200 scoring molecules were taken forward for docking with GNINA [60] into all the provided crystal structures. The molecules were ranked by their final mean affinities.

SID 85 used the provided database subset corresponding to 2, 5 and 2 number of hops, hac and rac, respectively. They justified that those parameters would provide a good balance between similarity to the co-crystallized compounds and increased molecular size of the follow-ups which is necessary to increase the number of favourable contacts. They further filtered out compounds that have a Dice Similarity over Morgan Fingerprints of the co-crystallized compounds smaller than 0.7. Finally, they removed compounds lacking large enough common substructures which resulted in a final set of > 8000 molecules. They then used SkeleDock [58] to generate follow-up poses. This software uses maximum common substructure constrained docking over provided protein-ligand structure which were, here, the co-crystallized fragments. The poses were scored with rDock [53] and the top 100 compounds were selected and manually inspected.

SID 86 used did not filter the database in their workflow. All compounds were generated from the provided smiles with CORINA [47] and protonated at physiological pH with Schrodinger LigPrep. Single docking poses were generated in GOLD and scored with ChemScore using a ligand efficiency of 30% (against 200% in Stage 1). The top 100 scoring compounds were selected without further filtering or validation.

SID 87 applied a custom filter based on common chemical descriptors to the whole database to select follow-up compounds. Selected compounds should have between 10 and 34 heavy atoms, more than 2 hydrogen bond donors, between 50 100% of the heavy atoms in a SP3 hybridization form, between 3 and 10 rotatable bonds, between 2 and 3 aromatic rings, 1 or more aromatic heterocycle, between 1 and 2 aliphatic rings, no more than 4 rings and a molecular weight between 360 and 440 Da. They further filtered out compounds containing amide bonds were excluded except tertiary amides and lactams. They built a pharmacophore in Molegro Virtual Docker [59] by merging the poses of F367, F389, F558 and F584 which cover all areas of the Kac binding site. The pharmacophore was used to apply template filtering to the selected compounds and the top 10% of those were selected. Finally, the poses were scored against the C2 apo crystal structure and the top 16 structures were submitted.

**Fig. 8:**
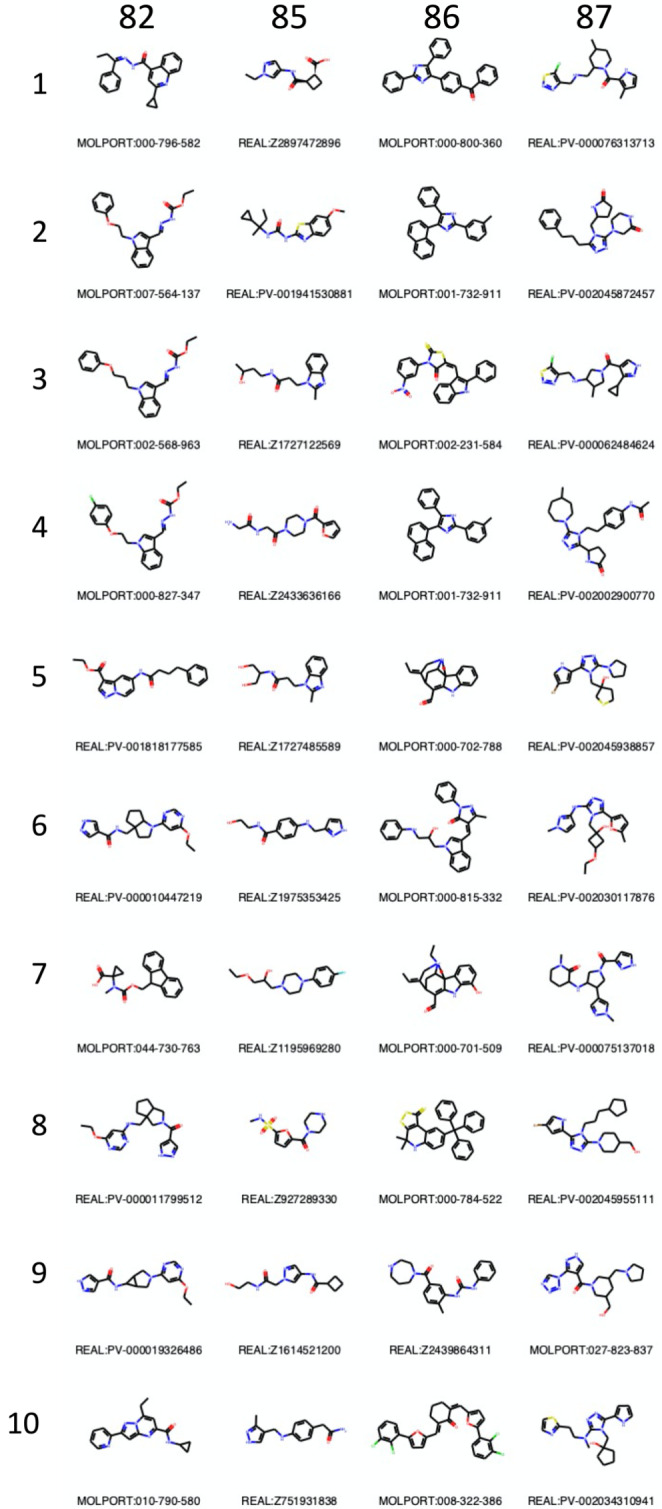
Chemical structures of the top 10 compounds submitted in Stage 3 follow-ups enumeration submissions. *The column and row labels indicate the SID and ranks, respectively.* The Molport or Enamine Real identifiers are shown below each 2D molecular representation. Th full rankings can be accessed on the github page of the challenge: https://github.com/samplchallenges/SAMPL7/tree/master/protein_ligand/Analysis/Analysis-outputs-stage3 under stage3_submission_collection.csv

Unsurprisingly, all submissions show an increase in all molecular descriptor counts (**SI** Fig. 4), with respect to the original Kac fragments, which is paired with an increase in molecular weight. Similarly, the distribution of Tanimoto coefficients for each submission are shifted toward 1 showing that the submitted follow-ups are more similar to each other than the native fragments are. The intrinsic molecular size bias of the Tanimoto coefficient may also participate in the shift. The larger the molecules the larger number of features present in the binary array and is reflected by a higher similarity [61]. The top 10 molecules for each submission are displayed in **Fig **8.

SID 82 increased the number of rotatable bonds and molecular weight. However, the other descriptors were only moderately raised. This implies that these molecules would be relatively flexible for a low number of H-bond donor and acceptors implying a high entropic penalty of binding for a low enthalpic compensation. Some molecules also showed a high degree of chemical similarity to each other with a Tanimoto coefficient higher than 0.8. All molecules composing the top 10 were, nonetheless, rule of 5 compliant. Molecules ranked 2 to 9 are to flexible to be selected for biophysical validation. Molecules ranked 1 and 10 could potentially have good scaffold sampling all areas of the binding site while maintaining reduced flexibility relatively to other compounds in that submission.

SID 85 increased relatively largely the H-bond donors and acceptors whilst maintaining the number of rings and molecular weight relatively low which had a positive and negative impact on the topological surface area and logP, respectively. Reducing hydrophobicity in that fashion may be a suitable strategy, however, PHIP2 and bromodomain in general have a hydrophobic binding cavity implying that more hydrophobic compounds are likely to have an increased affinity. In addition, some compounds still have low feature counts, which keeps them in a fragment-like category. The compounds are also relatively diverse when compared to other submissions and were all rule of 5 compliant. No molecules from that submission would have been selected for biophysical validation and they are too small and/ or too similar to the fragment hits and we would not expect them increase binding affinity significantly enough. For example, molecules 2, 3, 4, 5 and 7 are almost exactly similar to fragments 579, 217, 709, 217 and 710, respectively and the other submitted compounds remain smaller than the largest fragment.

SID 86 increased the most the number of rings without a similar increase in polar features in the follow-ups which resulted in large logP and molecular weight against a low topological polar surface area indicative of greasy compounds. Only 4 out of the top 10 were rule of 5 compliant. Although those compounds would probably sample well the predominantly hydrophobic binding site, they would aggregation problems in solution. Only compounds 5, 7 and 9 have an acceptable predicted logP. 5 and 7 are extremely similar to each other so one of the two would be selected along with compound 7 for further testing.

Finally, the molecules submitted by SID 87 have relatively large and varied numbers of H-bond donors and rotatable bonds while the count of ring and H-bond acceptors remain low and non-diverse. These designs result in a set of relatively high chemical similarity and were all rule of 5 compliant. They are almost all based on a central ring that expands into 3 different directions to sample the whole binding site. This appears to be suitable strategy, but care should be given to keeping molecules flexibility to a minimum. For example, molecules 2, 8 and 10 display a high number of rotatable bonds. Molecules 1 and 3 would not be able to sample the water cavity. Thus molecules 4, 5, 6 and 7 would have been selected for biophysical validation.

## Discussion

The application of computational methods to the design of fragment-based inhibitors is now an essential part of almost all fragment-based drug discovery pipelines. These computational methods are, however, better suited for larger, drug-like molecules and there is a general lack of prospective validation/evaluation in fragment space. Such evaluation and validation studies may identify pitfalls that could be later improved to ultimately lead to more efficient workflows in fragment-based drug discovery. To that end, a unique opportunity arising from our work on PHIP2 presented itself. To the best of our knowledge, no such inhibitor has been yet discovered against PHIP, despite it being implicated in cancers [27]. Our crystallographic fragment screening resulted in the identification of 52 binders across 4 sites (Fig. 2 A), 47 of which were bound to the pharmacologically relevant Kac binding site (**Fig **3). As such, this presented an ideal scenario to set up a SAMPL challenge to explore the capability of rapid computational tools for exploring fragment binding. Our screening campaign resulted in hit rates of 5.88% and 6.50% at the Kac binding site and across all sites, respectively. Crude analysis of our *in-house* data shows that hit rates at the Xchem vary between approximately 2% and 15% with an average of 7%, per protein target. Thus, our crystallographic results fall within expected hit rates. It must be remembered that there are caveats with the experimental approach – crystallization conditions and the constraints of the lattice may all be things that confound predictive technologies. Indeed, in the case of PHIP2, there are apo protein structures alternative to our C2 form in difference space groups, namely P212121 (PDB-ID: 7AV8) and P21212 (PDB-ID: 7BBO). Differences in the rotamer of a key threonine in the binding site can be observed. The P212121 structure was not deposited whilst the P21212 had associated fragment-bound structures in the PDB at the time of the challenge. Crystallographic space groups define the arrangement of protein units composing the crystal lattice. Thus, when proteins are crystallized in different space group the packing effects (such as contact points between units) can lead to structural variation. In this case, the PHIP2 Kac binding was not exempt of such effect and several residues display different arrangement between crystal forms. The precise effects on fragment screens are not fully known in this regard but one might expect some differences in fragment binding. Nevertheless, this scenario presents a very challenging scenario to test current high-throughput fragment docking approaches.

The challenge was initially divided into 3 stages: (1) virtual screening, (2) binding pose prediction and (3) enumeration of fragment follow-up compounds from a database. Virtual screening aims to identify molecules that bind to a target and can be important if such information is not available.

Stage 1 assessed the ability of submitted methods to discriminate crystallographic binders and non-binders. Excluding the null submission (SID:33), all submissions but one (SID: 37), used protein-ligand docking for virtual screening purposes. A variety of protocols were, however, submitted with techniques involved ranging in computational expense and complexity. For example, SID 38 used a plain docking protocol while SID 35 and 44 and used a more elaborated MD-based workflows (Table 1). Despite the variety of method employed, none managed to perform better than random, which highlights the difficulty of the task, but is also disappointing (Fig. 5). Some methods, however managed to apply the correct bias strategy. All submissions but 35 predicted a large excess of non-binders. which is in accordance with the experimental binder to non-binder ratio, a strategy that, despite being correct, reduces the probability of identifying true positives. Such strategy was effectively decided by the users by determining a cut-off that would bias the results in this fashion (Fig. 5). Another, challenging aspect of such a task was that the fragments have a limited range of descriptors (**SI** Fig. 2), which would ultimately make them similar in binding modes and therefore discrimination at the scoring stage would also be challenging [62].

Few studies have prospectively assessed virtual screening of both fragment-like and larger molecules. Notably, SAMPL3 focused on trypsin binders and built a dataset of 544 fragments against which some bound structures were resolved, and affinity measurements were collected by ITC and SPR [63]. 20 of these 544 fragments were considered as true binders. SAMPL3 virtual screening performance was better than with our PHIP2 fragments with a submission achieving an area under the curve of up to 0.8 [64] by employing a similar docking protocol to SID 52. SAMPL4 focused on fragment follow-ups that bind to the HIV integrase. They built a library around 4 fragments in an attempt to design a more potent inhibitor. This implies that the subsequent follow-ups share a similar core and therefore have some degree of chemical similarity [64]. Compounds that resolved with X-ray crystallography but did not bind better than 2 mM, based on SPR measurements, were classified as inactives. A total of 305 compounds, including 56 actives, were put forward for the challenge. 5 submissions out of 26 achieved an area under the curve equal or better than 0.6 which is, again better than our PHIP2 fragments [20]. The D3R grand challenges also blindly assess computational method in a similar fashion to SAMPL but many focus on affinity rankings of more drug-like compounds instead of a binary binder/non-binder classification. This makes the comparison of performances between these tasks harder because the D3R tasks fall in the category of binding free energy prediction [22, 65–67].

It is hard to precisely rationalise the poor results of the Stage 1 submissions. One notable difference between this task and SAMPL3 and SAMPL4 is that the classification was solely based on crystallographic binding. We emphasised this, when setting up the stage, by suggesting that participants should aim to reproduce the crystallographic screening results. This may be a more important consideration than first assumed as there is the possibility that our fragments do not necessarily bind in solution, whereas scoring functions are almost always calibrated and validated against solution and structural data [68]. Work is currently underway to ascertain in-solution binding affinities (via isothermal titration calorimetry). Similarly, MD based workflows tend to estimate solution like states, which of course will differ from a crystal state. This is, however, hard to generalise as docking protocol performances often vary between targets.

Stage 2 was a binding pose prediction exercise - a task that is more frequently assessed than virtual screenings. Only 5 ranked submissions were received for this stage of the challenge (Table 2). This surprisingly low number of participations was likely due to the short timeframe imposed; only 2 weeks (due to internal pressures on Stage 3). Although this stage was shorter than we would have liked, the small number of submissions highlights the lack of flexible, fast and fully automated workflows available. The 5 submissions received did not perform well, stressing the difficulty and potential scope for improvement regarding fast 3D binding pose prediction for fragments (Fig. 7). In addition, not all participants submitted 5 poses per ligand despite highlighting in the guidelines that they could do so (Fig. 6). The best performance was SID 77 and employed a biased MD-based protocol. This provides an example where protocols that treat the protein and water as flexible can outperform rigid dockings when trying to reproduce static crystallographic data. The second and third best performing methods docked the compounds into the provided apo structure. The two worst performing methods picked different fragment-bound receptors from the PDB. This shows that docking-based binding pose predictions of crystallographic fragments against a particular receptor conformation is highly sensitive to the choice of receptor conformation and that the presence of alternative ligand-bound structures does not always improve the prediction quality (Fig. 7). Another challenging aspect of docking to PHIP2, and bromodomains in general, is that only some fragments retain the conserved water network. This implies that the choice of retaining or removing these waters will have an adverse impact on some predictions. For example, SID 75, kept all structural waters in their protocol, which made impossible the correct prediction of water-displacing fragments such as F584 (Fig. 4). The importance of waters ligand binding and docking is however a known fact and has been described elsewhere [69, 70]. More advanced protocol such as Grand Canonical Monte Carlo analysis of water stabilities may also guide modellers in choosing which molecules should be kept in the receptor structure [44, 71].

SAMPL4 also assessed binding pose prediction of a similar number of ligands. This challenge had the additional difficulty of the target having several binding sites, which led to relatively high RMSD in cases where the incorrect site of a particular compound was predicted [20]. Similarly, to this challenge, only one third (3 out 15) of the submissions correctly predicted the binding pose at their given sites. The performance of these best submissions was overall better than the predictions presented here. Here most ligands were incorrectly predicted in all submissions as opposed to SAMPL3 where most ligands were correctly predicted in at least 1 submission, but this is likely due to the relatively higher number of submissions in SAMPL3. D3R grand challenges also assess blinded predictions of binding pose as well as other meaningful quantities to drug discovery. D3R grand challenge 4 submissions achieved excellent results for binding pose predictions of macrocycles against BACE1. The good performances were partially attributed to the presence of many ligand-bound crystal structures in the PDB which could guide docking results [22]. The most obvious difference with our data is the size of ligand, thus suggesting, that docking of larger ligand may be an easier task than smaller fragment-like molecules although correct sampling of the ligand conformation may present issues.

Future challenges may benefit from participants submitting the scores associated with different molecules and poses for virtual screening and binding pose prediction respectively. This would allow the investigation of the energy surface, for example by plotting screening rank or RMSD against docking score. For fragment-like molecules, we expect these slopes to be relatively shallow and noisey. Fragments typically have low potencies, in the µM to mM ranges, implying that their free energy surfaces are relatively flat with shallow minima associated with many alternative binding poses and therefore rendering discrimination much more difficult. This is especially true in solution where protein degrees of freedom are unrestrained (cf. the crystalline state, where lattice packing may be a factor). Lead and drug-like molecules generally have nM potencies implying that their free energy surfaces have more pronounced energy minima from which actives could more readily be discerned. SAMPL4 [72], D3R 2 [65] and D3R 4 [22] treated larger (non-fragment) molecules and binding pose predictions were generally much better, this lending support to the postulation above.

The Kac binding site being divided into four subsites (Fig. 2.) presents an additional level of complexity by further widening the energy landscape and rendering sampling more difficult when comparing to simpler binding sites such as that found in T4 lysozyme. Three of these subsites (namely the central void, the water cavity and the BC-interface) contain hydrophobic residues implying that search method must be able to differentiate between similar environments. Thus, correct identification and discrimination of the subsite(s) for particular fragments is essential in obtaining correct predictions and this may and will influence the prediction quality for virtual screening and binding pose predictions. Furthermore, the fragments also have similar physico-chemical characteristics (**SI** Fig. 2.) implying that precise methods will be needed to discriminate binders from non-binders.

Stage 3 of this challenge was atypical. It consisted in selecting compounds from a large database that would likely bind with increased potency to our PHIP2 target. The participants were provided with cocrystal structures and tasked to filter the database to select binders with a method of their choice. To the best of our knowledge, this was the first ever challenge of the kind and compounds would have been screened by X-ray crystallography at the XChem facility. Unfortunately, we did not proceed with this stage because of the COVID-19 pandemic. The participants were given 1 month to submit up to 100 molecules, and we received only 4 submissions which, like stage 2, indicates a lack of responsive workflow despite this unique opportunity to test follow-up enumeration methods in a truly prospective way (Table 3). One striking submission from the top 10 compounds was within SID 86 where the library was not filtered, and all compounds docked. This resulted in large molecules with larger logP values that in most cases violated the rule of 5 (**Fig **8). Such molecules would not be viable candidates for screening and would likely not have been tested experimentally even if COVID had not intervened, and as such illustrates that appropriate filtering of the library or post-filtering of the docking results is almost certainly necessary when such an approach is used in order to identify viable compounds. The other methods employed diverse forms of pre-filtering. In 2 cases the filtering criterions (based on chemical descriptors) were explicitly introduced while in one case the filtering was applied based on a machine learning selection process. 2 out of 4 submissions provided docking files, but in different formats, which prevented systematic analysis of these results. The workflows employed in this stage were quite computationally cheap compared to what is available and more routinely applied such MD-based screening or free energy calculations. Reviewing and contrasting common screening strategy falls beyond the scope of this work. A larger-scale but similar enterprise was the COVID-moonshot that crowdsourced an anti-COVID-19 MPro inhibitor from fragment screening data [20]. The organizers gathered hundreds of scientists who voluntarily submitted molecules which were synthesized and tested biophysically with the aim of producing an IP-free oral antiviral.

All stages of this SAMPL edition were clearly challenging. What have we learned from this? Most submissions did not directly aim to reproduce experimental results by mimicking crystallographic conditions and binding although this was strongly emphasised during the challenge. Chemical factors were not taken into consideration despite the detailed experimental protocol being provided. For example, some participants protonated the protein and ligands assuming a physiological pH of 7.4 although we highlighted that our PHIP2 crystals were grown around pH 4.6. No ligand-bound structural information with this C2 crystal form was available prior to the start of the challenge. Thus, participants employed fragment-bound PHIP2 structures crystalized in other space groups to guide their predictions. Given that using various ligand-bound structures showed improved predictions in previous challenges this was a sensible move. However, previous challenges considered larger, non-fragment molecules. In this challenge, using other fragment-bound structures appeared to have had a negative effect on binding pose predictions, likely, because experimental screening was performed by soaking the compounds onto already-made crystals. This implies that the proteins composing the lattice are already folded in a particular conformation, and thus prediction of binding to this particular crystal lattice may well be different from might be observed in other crystal forms or in solution. Overall, the binding site conformations between the C2 and P21212 crystal forms show the most variation in the binding site, at the BC- and ZA-loops (Fig. 1 A), where amino acids important in binding, such as tyrosine 1350 or threonine 1396 have different orientations. These differences would explain the impact on docking performances as they have a direct impact on binding site volume and interaction availabilities. Thus, we perceive that, in this case, using different fragment-bound crystals was a mistake and computational identification of crystal binders should be preferably performed against the targeted crystal form (which we did provide here as the apo-structure) (Fig. 7).

Another factor that may be important here is that scoring and rescoring methods are normally calibrated based on existing structures paired with affinity data, whereas our PHIP2 fragments benchmark was only based on crystallographic results, and we are presently unaware if these bind into solution. Thus, commonly used methods may not be able to detect nor reproduce such weak crystallographically observed events. Other target specific considerations, such as the important water networks, likely also participate in making this SAMPL7 challenge difficult. Overall, performance of those that did enter illustrates that this is difficult to apply computational predictions on fragments in a prospective way at least in the case of this particular target.

Despite the relatively poor performance of the predictions, this work illustrates that there is plenty of scope of improvement in this area. This also implies that experimental workflows are still heavily needed to identify crystallographic fragment binders. The small number of submissions received, particularly for Stage 2 and 3 also highlights the lack of responsive workflows although our short timescales were designed to reflect real-world conditions. Despite the relatively poor performance of in silico methods in this challenge, the work has highlighted key areas of development that researchers can focus on in the coming years and it should be remembered that there have been many other examples of where computational methods have made significant contributions to fragment-based drug design [73].

The potential avenues to improve such predictions may include better treatment of water molecules. This could be done by using MD-based methods, selecting strongly interacting water by Grand Canonical Monte Carlo analysis [44, 74] or creating an ensemble of receptors that include all possible combinations of water networks within and surrounding the binding site. Identification of relevant conformational changes may also be achieved via MD-based methods or flexible docking. Inclusion of crystal symmetry mates around the binding site may also improve prediction by mimicking more precisely crystal conditions. Fragments make few but usually “high-quality” interactions and thus an explicit stage that captures that might well improve confidence. This could be done via hotspot or pharmacophore rescoring/ selection of the poses for example. The creation of tailored scoring functions trained on fragments and or crystallographic data only may yield better results [75]. All of those improvements may also be applied to fragment follow-up selection, which would also benefit from better library filtering and mining tools.
